# Cost-effectiveness of liquid biopsy screening for gastric cancer in the United States

**DOI:** 10.1093/jncics/pkag090

**Published:** 2026-09-08

**Authors:** Jeong Yun Yang, Alice Agyekum, Kevin J Rouse, Sheila D Rustgi, Chin Hur

**Affiliations:** Division of Gastroenterology, Department of Medicine, NYU Grossman School of Medicine, New York, NY, United States; Department of Medicine, Columbia University Irving Medical Center, New York, NY, United States; Department of Medicine, Columbia University Irving Medical Center, New York, NY, United States; Department of Medicine, Columbia University Irving Medical Center, New York, NY, United States; Department of Medicine, Columbia University Irving Medical Center, New York, NY, United States

## Abstract

**Background:**

Despite the frequent late-stage diagnosis of gastric cancer (GC), the United States lacks a routine GC screening program. Liquid biopsy (LB), specifically blood-based tests, has emerged as a potential screening approach, but it is unclear whether such a strategy is cost-effective. We, therefore, evaluated the cost-effectiveness of LB screening for GC in the United States.

**Methods:**

A Markov model was developed to compare no screening (current standard-of-care) and 2 LB-screening strategies. The study used a simulated cohort of 45-year-old individuals with average risk of GC. Primary model outcomes included life expectancy, quality-adjusted life-years (QALYs), total cost, and incremental cost-effectiveness ratios (ICERs). A strategy was considered cost-effective if the ICER was < the US willingness-to-pay threshold of $100 000 per quality-adjusted-life-year gained.

**Results:**

In the base-case analysis, LB screening strategies were not cost-effective, with an ICER of $917 826 per QALY gained. Screening became cost-effective for high-risk subpopulations or when the per-test cost was approximately $89.

**Conclusions:**

This economic evaluation of LB for GC screening found that while LB strategies are effective in reducing cancer incidence and mortality, they are not cost-effective due to high test costs. Our findings could help inform the potential planning of future clinical validation studies for LB tests used for GC screening.

## Introduction

Gastric cancer (GC), predominantly noncardia gastric adenocarcinoma, remains a significant global health burden as one of the leading causes of cancer-related mortality worldwide.^1^ In the United States, GC is often diagnosed at an advanced stage, contributing to poor outcomes and a 5-year survival rate of 36%.^2^^,^^3^ In contrast to high-incidence countries such as South Korea and Japan, where population-wide endoscopic screening programs have demonstrably improved early detection and survival, no such screening guidelines exist in the United States due to the relatively low overall incidence of GC.^4^^,^^5^ Nevertheless, GC disproportionately affects specific subpopulations, including first-generation immigrants from high-incidence regions and racial and ethnic minority groups such as Black, Hispanic, Asian, and Native American populations.^6^

Current guidelines from the American Gastroenterological Association (AGA) and the American College of Gastroenterology do not endorse routine population-based screening. Instead, they recommend risk-based screening limited to individuals with identifiable high-risk features, such as family history, premalignant conditions, or immigration from endemic regions, using esophagogastroduodenoscopy (EGD) as the preferred diagnostic modality.^6^ However, the absence of a standardized screening program and the invasive nature and resource intensity of EGD present significant barriers to implementation and broad uptake.

Recent advances in liquid biopsy (LB) technology, a noninvasive diagnostic method that detects tumor-derived biomarkers in blood, offer a promising alternative for early detection of GC. These blood-based tests, including assays that detect circulating tumor DNA and miRNA biomarkers, have the potential to detect GC earlier in the disease course, potentially improving outcomes through timely intervention. Despite this promise, the clinical effectiveness and cost-effectiveness of LB screening in the US population remain uncertain.^7-9^

To address this evidence gap, we developed a state-transition Markov model to evaluate the health and economic outcomes of 2 LB screening strategies compared to no screening or the natural history (NH), the current standard-of-care, in an average-risk US population. This analysis aims to inform the potential role of LB as a scalable, noninvasive screening approach for GC and to guide future policy considerations for its integration into US clinical practice.

## Methods

### Institutional review statement

This study used publicly available data and did not involve human subjects; thus, institutional review board approval was not required. The study followed the Consolidated Health Economic Evaluation Reporting Standards (CHEERS) reporting guidelines for cost-effectiveness analysis.^10^

### Model overview

We developed a state-transition Markov model to assess the cost-effectiveness of LB testing for GC screening compared with NH based on the AGA Expert Review.^6^ We simulated a hypothetical cohort of individuals aged 45 years at average GC risk. Screening strategies involved testing every 3 years from ages 45 to 75. We selected age 45 to align with international GC screening practices (South Korea: age 40; Japan: age 50), and US colorectal cancer screening, facilitating opportunistic co-screening with colonoscopy.^4^^,^^11^^,^^12^ Screening ceased after 75 years, although individuals could still develop GC as they aged.

### NH model

The model’s NH component simulated disease progression from normal gastric mucosa through premalignant states, including chronic atrophic gastritis (CAG), intestinal metaplasia (IM), low-grade dysplasia (LGD), and high-grade dysplasia (HGD) to preclinical and clinically detected GC (Figure 1). The model used annual cycles, with individuals entering at age 20 and followed until age 100 or death.

**Figure 1.**
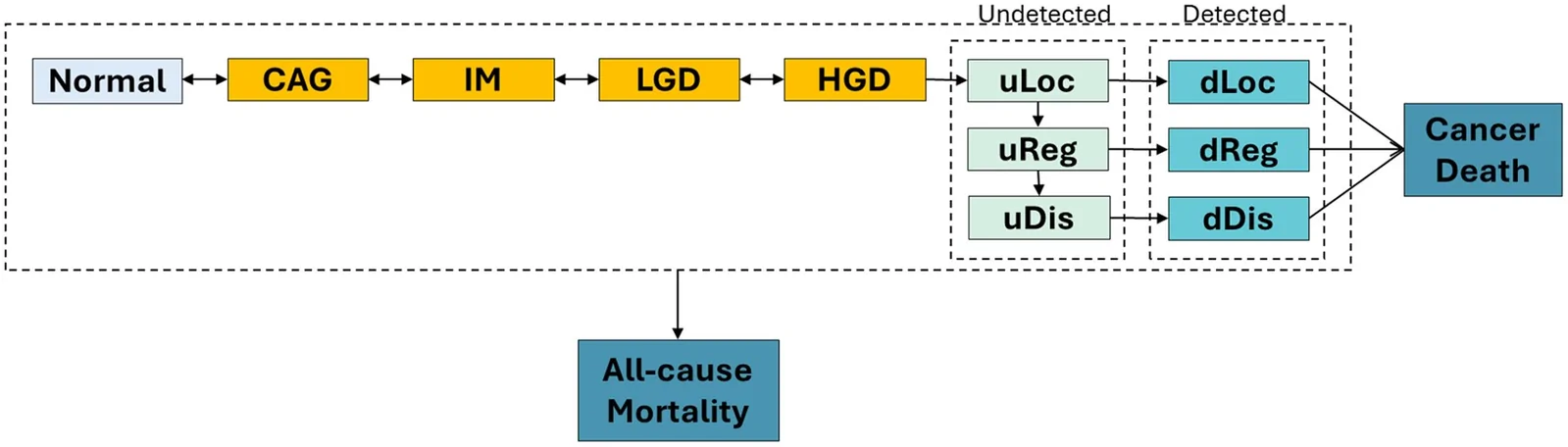
Model schematic of NH of GC. Abbreviations: CAG = chronic atrophic gastritis; Dis = distant gastric cancer; IM = intestinal metaplasia; LGD = low-grade dysplasia; HGD = high-grade dysplasia; Loc = local gastric cancer; Reg = regional gastric cancer.

Disease progression was stratified by cancer historic stage (local, regional, distant), with transition probabilities informed by published literature and model calibration (Table 1). Patients were allowed to regress between premalignant states. Preclinical GC could progress by stage or become symptomatically detected. Once diagnosed, patients remained in their detected cancer stage and were subject to stage- and age-specific GC mortality risks for up to 10 years, after which only all-cause mortality applied. Individuals could die from other causes at any age. All-cause mortality was obtained from US Centers for Disease Control and Prevention lifetables.^13^ GC incidence and mortality data were obtained from the Surveillance, Epidemiology, and End Results (SEER) 17 database (2000-2021).^14^ Because US-specific longitudinal data on premalignant transitions are limited and heterogeneous, initial pre-calibration transition rates were informed by previously published studies^16^^,^^18^^,^^19^^,^^28^ and then calibrated to the cumulative cancer incidence rate and stage distribution observed in SEER.

**Table 1.** Base-case model inputs and ranges used for sensitivity analyses.

Parameters	Base-case estimate	Range used in sensitivity analysis	PSA distribution	Sources
Age	20	n/a	n/a	
Probabilities				
All-cause mortality	lifetable	n/a	β	^13^
Local GC mortality	Table	n/a	β	^14^
Regional GC mortality	Table	n/a	β	^14^
Distant GC mortality	Table	n/a	β	^14^
**Test characteristics**				
EGD LGD sensitivity	0.51	0.3-0.7	β	^15^
EGD HGD sensitivity	0.74	0.6-0.85	β	^15^
EGD local GC sensitivity	0.78	0.6-0.85	β	^16^
EGD regional GC sensitivity	0.84	0.7-0.95	β	^16^
EGD distant GC sensitivity	0.9	0.8-1	β	^16^
LB-A local GC sensitivity	0.167	0.14-0.185	β	^17^
LB-A regional GC sensitivity	0.636	0.5-0.75	β	^17^
LB-A distant GC sensitivity	1	0.9-1	β	^17^
LB-A specificity	0.993	0.9-1	β	^17^
LB-B HGD sensitivity	0.6	0.4-1	β	^7^
LB-B local GC sensitivity	0.63	0.5-0.8	β	^7^
LB-B regional GC sensitivity	0.82	0.74-0.902	β	^7^
LB-B distant GC sensitivity	0.93	0.837-1	β	^7^
LB-B specificity	0.89	0.8-0.979	β	^7^
**Model inputs**				
Successful EET	0.905	0.45-1	β	^28^
EGD complication	0.001	0.003-0.009	β	^16^
EET complication	0.002	0.001-0.009	β	^16^
Death from EGD complication	0.0001	0.00005-0.00015	β	^16^
Death from EET complication	0.0001	0.002-0.006	β	^16^
Normal to CAG	0.025	0.0251-0.100	β	^28^
CAG to IM	0.02	0.019-0.032	β	^28^
IM to LGD	0.03	0.0309-0.057	Beta	^28^
LGD to HGD	0.01	0.01-0.016	Beta	Expert opinion
HGD to local GC	0.12	0.119-0.14	Beta	^16^
Local to regional GC	0.45	0.4317-0.47	Beta	^18^
Regional to distant GC	0.58	0.574-0.61	Beta	^18^
CAG to normal	0.009	0.0057-0.009	Beta	^19^
IM to CAG	0.02	0.0153-0.02	Beta	^19^
LGD to IM	0.10	0.0716-0.10	Beta	^19^
HGD to LGD	0.01	0.0083-0.01	Beta	Expert opinion
Symptomatic local GC	0.12	0.12-0.134	Beta	^28^
Symptomatic regional GC	0.28	0.2844-0.303	Beta	^28^
Symptomatic distant GC	0.3	0.3-0.35	Beta	^28^
**Utilities**				
Healthy	1	n/a	Beta	n/a
CAG	0.98	0.735-1	Beta	^20^
IM	0.98	0.735-1	Beta	^20^
Dysplasia	0.98	0.735-1	Beta	^20^
Undetected GC	0.98	0.735-1	Beta	^20^
Local GC	0.773	0.580-0.966	Beta	^21^
Regional GC	0.590	0.442-0.737	Beta	^21^
Distant GC	0.404	0.303-0.505	Beta	^21^
EGD/EET	0.7 (1 day)	0.35-1	Beta	^22^
EGD/EET complications	0.7 (1 week)	n/a	Beta	^22^
**Costs ($)**				
EGD	1199	599.5-2398	Gamma	^23^
EET	1813	906.5-3626	Gamma	^24^
Surgery	31 495	15 747.5-62 990	Gamma	^25^ ^,^ ^28^
LB	1500	50-2000	Gamma	^26^
First year local GC costs	79 057	39 528.5-158 114	Gamma	^25^ ^,^ ^27^
Continuing care costs of local GC	10 389	5194.5-20 778	Gamma	^25^ ^,^ ^27^
Final year local GC costs	106 483	53 241.5-212 966	Gamma	^25^ ^,^ ^27^
First year regional GC costs	108 457	54 228.5-216 914	Gamma	^25^ ^,^ ^27^
Continuing care costs of regional GC	8519	4259.5-17 038	Gamma	^25^ ^,^ ^27^
Final year regional GC costs	127 468	63 734-254 936	Gamma	^25^ ^,^ ^27^
First year distant GC costs	101 392	50 696-202 784	Gamma	^25^ ^,^ ^27^
Continuing care costs of distant GC	21 193	10 596.5-42 386	Gamma	^25^ ^,^ ^27^
Final year distant GC costs	126 637	63 318.5-253 274	Gamma	^25^ ^,^ ^27^

Abbreviations: CAG = chronic atrophic gastritis; EET = endoscopic eradication therapy; EGD = esophagogastroduodenoscopy; GC = gastric cancer; HGD = high-grade dysplasia; IM = intestinal metaplasia; LB-A = liquid biopsy with low sensitivity for local cancers and no sensitivity for high-grade dysplasia; LB-B = liquid biopsy with high sensitivity for local cancers and sensitivity for high-grade dysplasia; LGD = low-grade dysplasia. Cost in U.S. dollars.

### Screening strategies

We evaluated the effectiveness and cost-effectiveness of 2 LB screening strategies: liquid biopsy A (LB-A) every 3 years and liquid biopsy B (LB-B) every 3 years, modeled after commercially available tests such as Galleri (GRAIL, Inc.)^17^^,^^29^ and GASTROClear (MiRXES Pte Ltd).^7^^,^^30^ LB-A was defined as having high specificity for advanced GC but low sensitivity for early-stage disease. LB-B was defined as capable of detecting premalignant lesions and highly sensitive for detecting local GC. Both strategies assumed testing every 3 years from ages 45 to 75. Positive tests prompted confirmatory EGD, with 100% adherence in the base case. Consistent with convention in prior screening cost‑effectiveness analyses, full base‑case adherence was assumed to isolate test‑performance and cost effects on the incremental cost-effectiveness ratios (ICERs).^31^^,^^32^ Test characteristics, including sensitivity and specificity for detecting HGD and GC, were derived from published literature and company reports (Table 1). LB-A and LB-B performances were derived from case-control validation cohorts of Galleri and GASTROClear, respectively.^7^^,^^17^

Patients with confirmed LGD or HGD were assumed to undergo endoscopic eradication therapy (EET) of the lesion and transition back to the IM state. Confirmed cancer cases received stage-appropriate treatment, with survival modeled using SEER-derived age- and stage-specific survival curves.^33^

An endoscopic screening strategy was not evaluated because current US guidelines do not recommend population-based endoscopic GC screening for average-risk individuals.^6^ We also did not model surveillance of premalignant lesions such as gastric IM as there is no strong evidence of the optimal surveillance interval in the US population.^6^

Costs included direct medical expenses associated with LB testing, confirmatory diagnostics, GC treatment, and post-treatment care (Table 2). In the absence of Centers for Medicare and Medicaid Services reimbursement for GC LB tests, costs were assumed equal for both assays, informed by listed price of Food and Drug Administration-approved LB test for colon cancer (approximately $1500 per test).^26^ Cancer treatment costs were based on published US cost data and adjusted to 2025 dollars using the Consumer Price Index (Table 1).

**Table 2.** Base-case results for cost, effectiveness, and clinical outcomes of GC screening using LB.

Strategy	Total cost	Incremental	Effectiveness (QALYs)	Incremental effectiveness	**ICER** ^a^ **($/QALY)**	Life expectancy	GC Incidence	**% averted** ^c^	GC Mortality	**% averted** ^c^	Lifetime EGDs per
**NH**	**$407.39**	**cost ref**	**27.093**	**(QALYs) ref**	**ref**	**79.027**	**0.684%**	**ref**	**0.318%**	**ref**	**10007**
LB-A Q3Y	$5078.56	$4671.16	27.093	0.000	Abs. dominated^b^	79.028	0.686%	0.30%	0.306%	−3.63%	73
LB-B Q3Y	$5306.41	$4899.02	27.099	0.005	$917 826.22	79.047	0.389%	−43.13%	0.155%	−51.1%	1049

Abbreviations: Abs. Dominated = absolutely dominated; GC = gastric cancer; ICER = incremental cost-effectiveness ratio; LB-A = liquid biopsy with low sensitivity for local cancers and no sensitivity for high-grade dysplasia; LB-B = liquid biopsy with high sensitivity for local cancers and sensitivity for high-grade dysplasia; NH = natural history; QALY = quality-adjusted life-years; ref = reference.

Bolded strategies indicate optimal strategy at a WTP threshold of $100 000/QALY. Total cost, incremental cost, effectiveness, incremental effectiveness, ICER, and life expectancy results are displayed per person.

a ICER is calculated as incremental cost over incremental effectiveness, relative to the next costly, nondominated strategy.

b Absolutely dominated indicates that the strategy resulted in greater costs and few QALYs gained compared to another strategy.

c Percent averted is calculated relative to the NH scenario.

Effectiveness was measured in quality-adjusted life-years (QALYs). Utility weights ranged from 0 (death) to 1 (perfect health), with disutilities applied for EGD, EET, and associated complications (Table 1). False-positive results incurred costs and utilities from unnecessary confirmatory procedures. Both costs and QALYs were discounted at 3% annually, consistent with current consensus panel guidelines and recommendations.^34^

### Model outcomes

Primary outcomes included life expectancy, QALYs, total costs, and ICERs. The ICER represents the average incremental cost associated with 1 additional QALY compared with a comparator.


$$\mathrm{ICER}=\frac{\mathrm{Cost}\;\mathrm{of}\;\mathrm{Intervention}-\mathrm{Cost}\;\mathrm{of}\;\mathrm{Comparator}}{\mathrm{QALY}\;\mathrm{of}\;\mathrm{Intervention}-\mathrm{QALY}\;\mathrm{of}\;\mathrm{Comparator}}$$

Secondary outcomes included lifetime GC incidence, stage distribution at diagnosis, and GC-specific mortality.

### Statistical analyses

Analyses were conducted using TreeAge Pro Healthcare 2025 (TreeAge, Williamstown, MA). One-way deterministic sensitivity analyses evaluated the impact of key parameters on outcomes, including test performance, costs, GC incidence, and adherence rates. Model robustness was further assessed using probabilistic sensitivity analysis (PSA) with 100 000 Monte Carlo iterations, sampling costs from gamma distributions and test performance and utilities from beta distributions.^35-37^ Scenario analyses explored variations in GC incidence (2-fold and 10-fold the base-case rates) serving as a proxy for higher risk patient populations, and screening adherence rates ranging from 20% to 100%, to assess these important parameters’ effects on cost-effectiveness and clinical outcomes.

## Results

In the base-case analysis, screening with LB-A every 3 years yielded 0.001-year gain in life expectancy compared with NH at an additional cost of $4671.16, resulting in LB-A being dominated by the NH strategy (Table 2, **Figure S1**). Screening with LB-B every 3 years increased both life expectancy and QALYs compared with NH. Specifically, LB-B increased QALYs by 0.005 and life expectancy by 0.02 years per individual, at an incremental cost of $4899.02 per person, corresponding to an ICER of $917 826.22 per QALY gained.

Clinically, LB-A produced minimal reductions in GC incidence and mortality, consistent with its limited ability to detect premalignant lesions and early-stage disease. In contrast, LB-B reduced lifetime GC incidence by 43% (0.389% vs 0.684% under NH) and GC mortality by 51% (0.155% vs 0.318%). Both LB strategies resulted in higher lifetime EGDs per 1000 persons (73 and 1049 for LB-A and LB-B, respectively) compared with NH (7 lifetime EGDs per 1000 persons) (Table 2).

Compared with NH, LB-A modestly but favorably shifted the stage distribution at diagnosis, with fewer cancers detected at distant stage (32% vs 40%) and more detected at local (31% vs 27%) and regional (37% vs 33%) stages (Table 3). The stage shift was more pronounced with LB-B, which substantially increased the proportion of cancers detected at local stage (42%) and reduced the proportion at distant stage (25%) relative to NH.

**Table 3.** Stage shift in GC diagnosis with LB screening compared to no screening.

Stage shift	Local	Regional	Distant
NH	27%	33%	40%
LB-A	31%	37%	32%
LB-B	42%	33%	25%

Abbreviations: LB-A = liquid biopsy with low sensitivity for local cancers and no sensitivity for high-grade dysplasia; LB-B = liquid biopsy with high sensitivity for local cancers and sensitivity for high-grade dysplasia; NH = natural history.

One-way sensitivity analyses identified key drivers of model outcomes. For LB-A, the model was most sensitive to EGD sensitivity for detecting distant and local GC (**Figure S2**). Regardless of the cost, LB-A was never cost-effective (**Figure S2**). For LB-B, cost was the primary driver (**Figure S3**). For LB-B to be cost-effective, the cost would need to be reduced to approximately $89 per test.

Scenario analyses explored higher GC incidence by 2- and 10-folds to analyze cost-effectiveness in high incidence subpopulations, such as American Indian and Alaska Natives (2-fold compared to non-Hispanic Whites)^38^ and Korean Americans (>10-fold compared to non-Hispanic Whites).^39^ At 2-fold GC incidence, NH remained the cost-effective strategy, as LB-B yielded only a modest QALY gain of 0.011 at an ICER of $423 220 per QALY. At 10-fold GC incidence, the LB-B strategy became cost-effective, with an ICER of $51 486 per QALY gained. Under this scenario, LB-B prevented nearly 42% of GC cases (3.98% vs 6.84%) and approximately 49% of GC deaths (1.75% vs 3.41%), while increasing lifetime EGDs to 1108 per 1000 individuals. Across all scenarios, LB-A remained dominated with negligible impact on outcomes (Table 4).

**Table 4.** Sensitivity analysis varying GC incidence.

Strategy	Total cost	Incrementalcost	Effectiveness (QALYs)	Incremental effectiveness (QALYs)	**ICER** ^a^ **($/QALY)**	Life expectancy	GC incidence	**% averted** ^c^	GC mortality	**% averted** ^c^	Lifetime EGDs per 1000
**1× incidence (base-case)**											
**NH**	**$407.39**	**ref**	**27.093**	**ref**	**ref**	**79.027**	**0.684%**	**ref**	**0.318%**	**ref**	**7**
LB-A Q3Y	$5078.56	$4671.16	27.093	0.000	Abs. dominated^b^	79.028	0.686%	0.30%	0.306%	−3.63%	73
LB-B Q3Y	$5306.41	$4899.02	27.099	0.005	$917 826.22	79.047	0.389%	−43.1%	0.155%	−51.1%	1049
**2× incidence**											
**NH**	**$827.85**	**ref**	**27.055**	**ref**	**ref**	**78.985**	**1.37%**	**ref**	**0.64%**	**ref**	**14**
LB-A Q3Y	$5496.75	$4668.90	27.055	0.000	Abs. Dominated^b^	78.987	1.37%	0.31%	0.62%	−3.62%	80
LB-B Q3Y	$5550.93	$4723.08	27.066	0.011	$423 219.72	79.025	0.78%	−43.2%	0.31%	−51.1%	1056
**10× incidence**											
NH	$4623.67	ref	26.751	ref	ref	78.587	6.84%	ref	3.41%	ref	68
LB-A Q3Y	$9226.98	$1364.37	26.752	−0.062	Abs. Dominated^b^	78.602	6.84%	0.01%	3.28%	−3.87%	137
**LB-B Q3Y**	**$7862.61**	**$3238.94**	**26.814**	**0.063**	**$51 485.87**	**78.807**	**3.98%**	−**41.8%**	**1.75%**	−**48.8%**	**1108**

Abbreviations: Abs. Dominated = absolutely dominated; EGD = esophagogastroduodenoscopy; GC = gastric cancer; QALY = quality-adjusted life-years; ICER = incremental cost-effectiveness ratio; LB-A = liquid biopsy with low sensitivity for local cancers and no sensitivity for high-grade dysplasia; LB-B = liquid biopsy with high sensitivity for local cancers and sensitivity for high-grade dysplasia; NH = natural history; ref = reference.

Bolded strategies indicate optimal strategy at a WTP threshold of $100 000/QALY. Total cost, incremental cost, effectiveness, incremental effectiveness, ICER, and life expectancy results are displayed per person.

a ICER is calculated as incremental cost over incremental effectiveness, relative to the next costly, nondominated strategy.

b Absolutely dominated indicates that the strategy resulted in greater costs and few QALYs gained compared to another strategy.

c Percent averted is calculated relative to the NH scenario.

To account for varying real-world adherence rates, scenario analyses were conducted at 20%, 40%, 60%, and 80% adherence to screening for the base-case analysis. As adherence decreased from 100% to 20%, the effectiveness of LB-B declined modestly, with QALY gains ranging from 0.005 at 100% adherence to 0.001 at 20% adherence. Total costs and lifetime EGDs also decreased with lower adherence, from $5306 and 1049 EGDs per 1000 individuals at full adherence to $1387 and 215 EGDs per 1000 at 20% adherence. LB-A remained dominated under all adherence scenarios (**Table S1**).

We further evaluated the impact of LB specificity on cost-effectiveness and clinical outcomes (**Table S2**). For both LB-A and LB-B, lower specificity substantially increased lifetime EGDs and costs, while reducing effectiveness due to excess false positives. At 0% specificity, lifetime EGDs exceeded 9400 per 1000 individuals with no meaningful QALY gain. With higher specificity, both costs and lifetime EGDs declined markedly.

Additionally, we performed 1-way sensitivity analyses of LB performance characteristics. As the local cancer detection sensitivity increased, LB-A led to more detection of preclinical GC, resulting in higher GC incidence and lower GC mortality (**Table S3**). LB-B also resulted in higher GC incidence and lower GC mortality with higher local cancer detection sensitivity. Furthermore, increasing HGD detection sensitivity markedly improved LB-B outcomes (**Table S4**). At 0% HGD sensitivity, LB-B reduced GC incidence by 5% and mortality by 16%; at 100%, reductions reached 57% and 65%, respectively.

Two-way sensitivity analysis varying both specificity and HGD sensitivity (**Table S5**) showed the greatest reduction in GC incidence (−74%) and mortality (−78%) at 0% specificity and 100% HGD sensitivity, albeit with more than 9000 lifetime EGDs per 1000 individuals. As specificity increased, procedure burden and costs declined, but mortality benefit from higher HGD sensitivity persisted.

Probabilistic sensitivity analysis indicated that at a willingness-to-pay (WTP) threshold of $100 000, NH was the preferred strategy in almost 99.97% of iterations (Figure 2). At a WTP threshold of $1.5 million, LB-B was the preferred strategy in approximately 60% of iterations, while LB-A was never favored.

**Figure 2.**
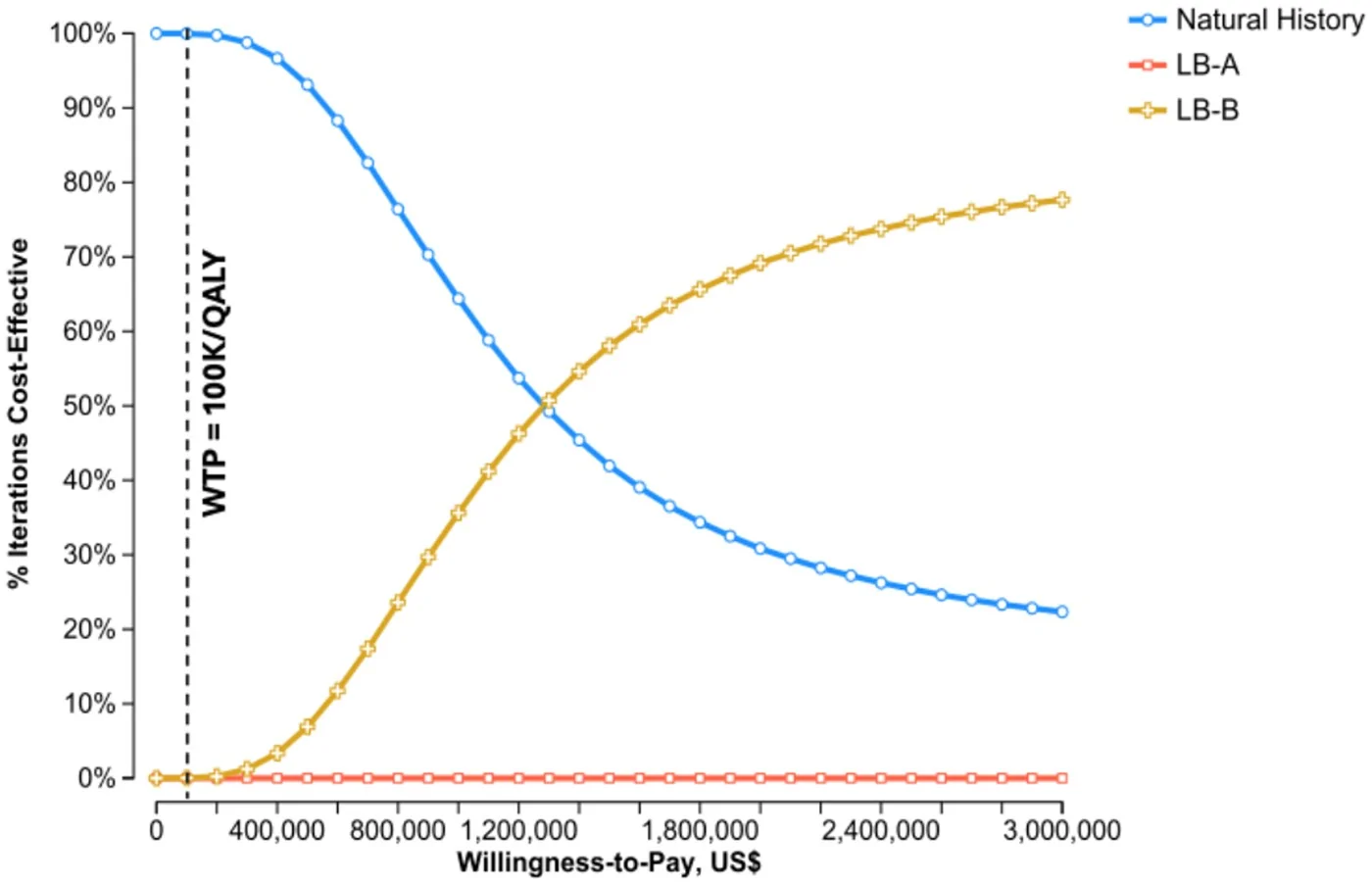
Probabilistic sensitivity analysis. Cost-effectiveness acceptability curve. Abbreviations: LB-A = liquid biopsy with low sensitivity for local cancers and no sensitivity for high-grade dysplasia; LB-B = liquid biopsy with high sensitivity for local cancers and sensitivity for high-grade dysplasia; WTP = willingness-to-pay. Probabilistic sensitivity analysis was conducted using 100 000 Monte-Carlo simulations. At the base-case WTP threshold of $100 000 per QALY, the NH strategy was preferred in nearly 100% of simulations.

## Discussion

This is the first cost-effectiveness analysis of LB screening for GC. Using a Markov model, we found that LB-B every 3 years provides modest gains in QALYs and reduces GC incidence and mortality compared with no screening in an average-risk US population; however, it is not cost-effective under current test performance and pricing. LB-A was not cost-effective because it provided a minimal gain in life expectancy at a high cost. These findings suggest that, while LB has potential as a noninvasive screening modality, its clinical utility depends heavily on test performance and cost.

Importantly, the scenario analyses highlight that the value of LB screening is substantially higher among groups with elevated GC risk. At GC incidence 10-fold higher than the general US population, which is consistent with risks observed in certain racial/ethnic subgroups such as Korean Americans, the LB-B strategy became cost-effective (ICER $51 485.87).^39^ This suggests that LB screening may be a viable, targeted strategy in high-risk subpopulations. Even among groups with a 2-fold GC incidence, LB-B screening resulted in meaningful GC prevention and reduction of GC mortality despite not crossing the cost-effectiveness threshold. These findings underscore the importance of risk stratification and support a targeted strategy rather than population-wide screening.

Other groups may also meet or exceed such elevated risk thresholds used in our high-incidence scenarios. These groups include individuals with autoimmune metaplastic atrophic gastritis (RR = 11.1; 95% CI = 6.39 to 19.1),^40^ advanced atrophic gastritis and IM based on Operative Link histopathologic staging systems routinely used in Europe and Latin America (OLGA/OLGIM) (RR = 27.7; 95% CI = 3.75 to 205),^41^ and hereditary syndromes (eg, CDH1 Peutz-Jeghers and Lynch syndrome).^42^ However, we did not model these specific conditions with varying lifetime GC risk separately. Because the OLGA/OLGIM staging system is not used in the United States and the NH of autoimmune metaplastic atrophic gastritis and hereditary syndromes differ from sporadic GCs, dedicated modeling is required; however, our scenario analyses suggest that LB screening could be valuable in these groups.

Our findings parallel cost-effectiveness analyses of LB screening for other cancers, such as colorectal cancer, where both cost and the ability to detect premalignant lesions critically determine value.^31^^,^^32^ The sensitivity analyses demonstrated that improvements in HGD and local GC sensitivities translated into significant reductions in GC incidence and mortality. Current assays with high sensitivity for detecting premalignant gastric lesions and early cancers, such as LB-B, may offer a cost-effective approach to improving GC outcomes if the cost is reduced by approximately 95% ($1500-$89). Conversely, reductions in specificity greatly inflated costs and procedure burden, with lifetime EGDs exceeding 9000 per 1000 individuals at 0% specificity. These tradeoffs emphasize that optimizing both specificity and sensitivity is critical to realizing the clinical and economic potential of LB screening.

Beyond cost considerations, LB screening would substantially increase demand for confirmatory endoscopies, as both true and false positive test results would require diagnostic follow-up. LB-B screening would require 1049 lifetime EGDs per 1000 individuals compared to only 7 per 1000 under current practice, a 150-fold increase in procedures (Table 2). Even in high-incidence populations where screening becomes cost-effective, this burden increases to 1108 EGDs per 1000 individuals. Current gastroenterologist shortages and capacity constraints in many US regions, particularly rural areas, raise questions about the feasibility of implementing such screening programs without significant increases in endoscopic capacity and associated healthcare costs.^43^ Bundling EGD with colonoscopy may partially mitigate these capacity constraints, as combined procedures minimally increase procedure time and reduce sedation costs.^44^^,^^45^

Limitations include uncertainty in test characteristics, which were derived from validation cohorts rather than prospective asymptomatic screening studies. Furthermore, the study assumed full adherence in the base case. Both limitations were addressed extensively in sensitivity analyses. Another limitation includes the cost of the tests estimated based on current commercially available LB tests for colorectal cancer as there is currently no LB test for GC that is reimbursed in the United States. However, sensitivity analysis varying the cost of LB found that without a substantial test price reduction, the conclusions remain unchanged. Additionally, LB-A is designed as a multi-cancer early detection test, and our GC-only model may, therefore, underestimate its overall cost-effectiveness. Exclusion of an EGD-based screening arm reflects current US guidelines, which do not recommend routine endoscopic screening for average-risk individuals or surveillance of precursor lesions such as gastric IM.

Finally, while our analysis is based on US population-level data, the results may have broader implications for regions with intermediate or high GC incidence. In such settings, LB may represent a promising strategy to expand access to noninvasive screening, particularly in communities where endoscopy is limited by cost, availability, or patient acceptance. Future work should explore real-world test performance, longitudinal outcomes, and integration with existing surveillance strategies to determine the optimal role of LB in GC prevention. Additionally, our high-incidence scenarios are approximations that do not capture the ethnicity-specific incidence trajectories or distinct NH of autoimmune or hereditary risk groups. Accordingly, the utility of LB screening in high-risk individuals, using NH models tailored to these groups, should be evaluated. Such efforts will be essential to determine whether LB can play a meaningful role in GC prevention in high-risk populations.

In summary, LB screening for GC at current test performance and estimated cost is not cost-effective in the average-risk US population. However, with substantial reductions in test cost and improvements in assay performance, particularly for early and premalignant lesion detection, LB screening could become a viable tool for early cancer detection and prevention in the average risk population in addition to subpopulations at high risk. Prospective studies incorporating clinical risk stratification and real-world implementation data are warranted before widespread adoption.

## Supplementary Material

pkag090_Supplementary_Data

## Data Availability

This study used only publicly available data from Centers for Disease Control and Prevention, Surveillance Epidemiology and End Results Program, and published literature. No individual patient data were used. Model parameters are detailed in the manuscript and Supplementary Materials. All analyses were conducted using TreeAge Pro Healthcare 2025 (TreeAge, Williamstown, MA), which requires licensed software. Analytic code and model specifications are available from the corresponding author upon request.
