# Supplementary material for: Cost-effectiveness of liquid biopsy screening for gastric cancer in the United States
Source: JNCI Cancer Spectr. 2026 Sep 8;10(5):pkag090. doi: 10.1093/jncics/pkag090 (PMC13651692; doi:10.1093/jncics/pkag090)
Supplement: Supplementary file 1 [file pkag090_supplementary_data.docx]

**Supplementary Tables and Figures**

1. **Figure S1. Cost-effectiveness plane of gastric cancer screening using liquid biopsy.**
2. **Figure S2. One-way sensitivity analysis. Tornado Diagram: Natural history vs LB-A.**
3. **Figure S3. One-way sensitivity analysis. Tornado Diagram: Natural history vs LB-B.**
4. **Table S1. Sensitivity analysis of initial adherence to liquid biopsy screening.**
5. **Table S2. One-way sensitivity analysis of liquid biopsy specificity.**
6. **Table S3. One-way sensitivity analysis of liquid biopsy sensitivity for detecting local cancer.**
7. **Table S4. One-way sensitivity analysis of HGD sensitivity for LB-B.**
8. **Table S5. Two-way sensitivity analysis varying liquid biopsy specificity and HGD detection.**

**Figure S1. Cost-effectiveness plane of gastric cancer screening using liquid biopsy.**

Abbreviations: LB-A (liquid biopsy with low sensitivity for local cancers and no sensitivity for high-grade dysplasia); LB-B (liquid biopsy with high sensitivity for local cancers and sensitivity for high-grade dysplasia); WTP (willingness-to-pay); QALY (quality-adjusted life-year).

Note: Costs and QALYs are presented as per-person estimates over the modeled time horizon. Strategies on the cost-effectiveness frontier (NH, LB-B) represent the most efficient options. LB-A is absolutely dominated, yielding higher costs and fewer QALYs than the alternatives.

**Figure S2. One-way sensitivity analysis. Tornado Diagram: Natural history vs LB-A.**

Abbreviations: GC (gastric cancer); LGD (low-grade dysplasia); HGD (high-grade dysplasia; EGD (esophagogastroduodenoscopy); LB-A (liquid biopsy with low sensitivity for local cancers and no sensitivity for high-grade dysplasia); WTP (willingness-to-pay).

Note: Parameters for test performance and costs were varied independently for the two liquid biopsy tests; those with the greatest impact on the ICER appear at the top, and those with the least impact at the bottom.

**Figure S3. One-way sensitivity analysis. Tornado Diagram: Natural history vs LB-B.**

Abbreviations: GC (gastric cancer); LGD (low-grade dysplasia); HGD (high-grade dysplasia; EGD (esophagogastroduodenoscopy); LB-B (liquid biopsy with high sensitivity for local cancers and sensitivity for high-grade dysplasia); WTP (willingness-to-pay).

Note: Parameters for test performance and costs were varied independently for the two liquid biopsy tests; those with the greatest impact on the ICER appear at the top, and those with the least impact at the bottom.

**Table S1. Sensitivity analysis of initial adherence to liquid biopsy screening.**

| **Strategy** | **Total Cost** | **Incremental Cost** | **Effectiveness (QALYs)** | **Incremental Effectiveness (QALYs)** | **ICER^a^ ($/QALY)** | **Life expectancy** | **GC Incidence** | **% averted^c^** | **GC Mortality** | **% averted^c^** | **Lifetime EGDs per 1000** |
| --- | --- | --- | --- | --- | --- | --- | --- | --- | --- | --- | --- |
| **20% adherence** | | | | | | | | | | | |
| **NH** | **$407.39** | **ref** | **27.093** | **ref** | **ref** | **79.027** | **0.684%** | **ref** | **0.318%** | **ref** | **7** |
| LB-A Q3Y | $1,341.62 | $934.23 | 27.093 | 0.000 | Abs. Dominated^b^ | 79.027 | 0.684% | 0.06% | 0.315% | -0.73% | 20 |
| LB-B Q3Y | $1,387.19 | $979.80 | 27.094 | 0.001 | $917,826.22 | 79.031 | 0.625% | -8.63% | 0.285% | -10.2% | 215 |
| **40% adherence** | | | | | | | | | | | |
| **NH** | **$407.39** | **ref** | **27.093** | **ref** | **ref** | **79.027** | **0.684%** | **ref** | **0.318%** | **ref** | **7** |
| LB-A Q3Y | $2,275.86 | $1,868.47 | 27.093 | 0.000 | Abs. Dominated^b^ | 79.027 | 0.685% | 0.12% | 0.313% | -1.45% | 33 |
| LB-B Q3Y | $2,367.00 | $1,959.61 | 27.095 | 0.002 | $917,826.22 | 79.035 | 0.566% | -17.3% | 0.253% | -20.5% | 424 |
| **60% adherence** | | | | | | | | | | | |
| **NH** | **$407.39** | **ref** | **27.093** | **ref** | **ref** | **79.027** | **0.684%** | **ref** | **0.318%** | **ref** | **7** |
| LB-B Q3Y | $3,210.09 | $2,802.70 | 27.093 | 0.000 | Abs. Dominated^b^ | 79.028 | 0.685% | 0.18% | 0.311% | -2.18% | 47 |
| LB-A Q3Y | $3,346.80 | $2,939.41 | 27.096 | 0.003 | $917,826.22 | 79.039 | 0.507% | -25.9% | 0.220% | -30.7% | 632 |
| **80% adherence** | | | | | | | | | | | |
| **NH** | **$407.39** | **ref** | **27.093** | **ref** | **ref** | **79.027** | **0.684%** | **ref** | **0.318%** | **ref** | **7** |
| LB-B Q3Y | $4,144.32 | $3,736.93 | 27.093 | 0.000 | Abs. Dominated^b^ | 79.028 | 0.686% | 0.24% | 0.309% | -2.91% | 60 |
| LB-A Q3Y | $4,326.60 | $3,919.21 | 27.097 | 0.004 | $917,826.22 | 79.043 | 0.448% | -34.5% | 0.188% | -40.9% | 841 |
| **100% adherence (base-case)** | | | | | | | | | | | |

**Table S1. Sensitivity analysis of initial adherence to liquid biopsy screening (continued).**

| **Strategy** | **Total Cost** | **Incremental Cost** | **Effectiveness (QALYs)** | **Incremental Effectiveness (QALYs)** | **ICER^a^ ($/QALY)** | **Life expectancy** | **GC Incidence** | **% averted^c^** | **GC Mortality** | **% averted^c^** | **Lifetime EGDs per 1000** |
| --- | --- | --- | --- | --- | --- | --- | --- | --- | --- | --- | --- |
| **NH** | **$407.39** | **ref** | **27.093** | **ref** | **ref** | **79.027** | **0.684%** | **ref** | **0.318%** | **ref** | **7** |
| LB-B Q3Y | $5,078.56 | $4,671.16 | 27.093 | 0.000 | Abs. Dominated^b^ | 79.028 | 0.686% | 0.30% | 0.306% | -3.63% | 73 |
| LB-A Q3Y | $5,306.41 | $4,899.02 | 27.099 | 0.005 | $917,826.22 | 79.047 | 0.389% | -43.1% | 0.155% | -51.1% | 1049 |

Abbreviations: GC (gastric cancer); QALY (quality-adjusted life-years); ICER (incremental cost-effectiveness ratio); NH (natural history); LB-A (liquid biopsy with low sensitivity for local cancers and no sensitivity for high-grade dysplasia); LB-B (liquid biopsy with high sensitivity for local cancers and sensitivity for high-grade dysplasia); Abs. Dominated (absolutely dominated); ref (reference); EGD (esophagogastroduodenoscopy).

Note: Bolded strategies indicate optimal strategy at a willingness-to-pay threshold of $100,000/QALY. Total cost, incremental cost, effectiveness, incremental effectiveness, incremental cost-effectiveness ratio, and life expectancy results are displayed per person.

^a^ICER is calculated as incremental cost over incremental effectiveness, relative to the next costly, nondominated strategy.

^b^Absolutely dominated indicates that the strategy resulted in greater costs and few QALYs gained compared to another strategy.

^c^Percent averted is calculated relative to the base-case natural history scenario.

**Table S2. One-way sensitivity analysis of liquid biopsy specificity.**

| **Specificity Level %** | **Total Cost** | **QALYs** | **Life expectancy** | **GC Incidence** | **% averted^a^** | **GC Mortality** | **% averted^a^** | **Lifetime EGDs per 1000** |
| --- | --- | --- | --- | --- | --- | --- | --- | --- |
| **LB-A** | | | | | | | | |
| 0 | $8,619.26 | 27.099 | 79.055 | 0.179% | -73.88% | 0.076% | -76.17% | 9466 |
| 20 | $7,877.74 | 27.099 | 79.052 | 0.224% | -67.27% | 0.097% | -69.54% | 7572 |
| 40 | $7,145.25 | 27.098 | 79.049 | 0.287% | -58.08% | 0.126% | -60.36% | 5679 |
| 60 | $6,425.91 | 27.097 | 79.044 | 0.376% | -45.05% | 0.167% | -47.49% | 3787 |
| 80 | $5,726.09 | 27.096 | 79.037 | 0.504% | -26.25% | 0.225% | -29.14% | 1896 |
| 100 | $5,055.74 | 27.093 | 79.028 | 0.694% | 1.47% | 0.310% | -2.52% | 7 |
| **LB-B** | | | | | | | | |
| 0 | $8,652.79 | 27.098 | 79.053 | 0.233% | -65.98% | 0.096% | -69.91% | 9462 |
| 20 | $7,898.34 | 27.099 | 79.052 | 0.258% | -62.21% | 0.106% | -66.75% | 7571 |
| 40 | $7,145.08 | 27.099 | 79.051 | 0.289% | -57.80% | 0.117% | -63.09% | 5679 |
| 60 | $6,393.26 | 27.099 | 79.049 | 0.324% | -52.59% | 0.131% | -58.80% | 3789 |
| 80 | $5,643.23 | 27.099 | 79.047 | 0.367% | -46.35% | 0.147% | -53.73% | 1899 |
| 100 | $4,895.42 | 27.098 | 79.045 | 0.419% | -38.77% | 0.166% | -47.65% | 10 |

Abbreviations: GC (gastric cancer); QALY (quality-adjusted life-years); LB-A (liquid biopsy with low sensitivity for local cancers and no sensitivity for high-grade dysplasia); LB-B (liquid biopsy with high sensitivity for local cancers and sensitivity for high-grade dysplasia); EGD (esophagogastroduodenoscopy).

^a^Percent averted is calculated relative to the base-case natural history scenario.

**Table S3. One-way sensitivity analysis of liquid biopsy sensitivity for detecting local cancer.**

| **Local Cancer Sensitivity Level %** | **Total Cost** | **QALYs** | **Life Expectancy** | **GC Incidence** | **% averted^a^** | **GC Mortality** | **% averted^a^** | **Lifetime EGDs per 1000** |
| --- | --- | --- | --- | --- | --- | --- | --- | --- |
| **LB-A** | | | | | | | | |
| 0 | $5,078.34 | 27.093 | 79.026 | 0.684% | -0.01% | 0.31% | -0.96% | 73 |
| 20 | $5,078.60 | 27.093 | 79.028 | 0.686% | 0.35% | 0.30% | -4.15% | 73 |
| 40 | $5,078.85 | 27.094 | 79.030 | 0.689% | 0.70% | 0.29% | -7.23% | 74 |
| 60 | $5,079.10 | 27.094 | 79.031 | 0.691% | 1.04% | 0.29% | -10.20% | 74 |
| 80 | $5,079.34 | 27.094 | 79.033 | 0.693% | 1.36% | 0.28% | -13.07% | 74 |
| 100 | $5,079.57 | 27.095 | 79.034 | 0.695% | 1.67% | 0.27% | -15.85% | 74 |
| **LB-B** | | | | | | | | |
| 0 | $5,305.74 | 27.098 | 79.044 | 0.385% | -43.66% | 0.171% | -46.08% | 1049 |
| 20 | $5,305.96 | 27.098 | 79.045 | 0.387% | -43.48% | 0.166% | -47.75% | 1049 |
| 40 | $5,306.17 | 27.098 | 79.046 | 0.388% | -43.31% | 0.161% | -49.35% | 1049 |
| 60 | $5,306.38 | 27.099 | 79.046 | 0.389% | -43.15% | 0.156% | -50.91% | 1049 |
| 80 | $5,306.58 | 27.099 | 79.047 | 0.390% | -42.99% | 0.151% | -52.40% | 1049 |
| 100 | $5,306.77 | 27.099 | 79.048 | 0.391% | -42.84% | 0.147% | -53.85% | 1049 |

Abbreviations: GC (gastric cancer); QALY (quality-adjusted life-years; LB-A (liquid biopsy with low sensitivity for local cancers and no sensitivity for high-grade dysplasia); LB-B (liquid biopsy with high sensitivity for local cancers and sensitivity for high-grade dysplasia); EGD (esophagogastroduodenoscopy).

^a^Percent averted is calculated relative to the base-case natural history scenario.

**Table S4. One-way sensitivity analysis of HGD sensitivity for LB-B.**

| **HGD Sensitivity Level %** | **Total Cost** | **QALYs** | **Life Expectancy** | **GC Incidence** | **% averted^a^** | **GC Mortality** | **% averted^a^** | **Lifetime EGDs per 1000** |
| --- | --- | --- | --- | --- | --- | --- | --- | --- |
| **LB-B** | | | | | | | | |
| 0 | $5,455.30 | 27.094 | 79.033 | 0.649% | -5.05% | 0.267% | -16.13% | 1045 |
| 20 | $5,393.76 | 27.096 | 79.039 | 0.540% | -21.10% | 0.220% | -30.73% | 1047 |
| 40 | $5,345.34 | 27.097 | 79.043 | 0.455% | -33.42% | 0.184% | -42.09% | 1048 |
| 60 | $5,306.41 | 27.099 | 79.047 | 0.389% | -43.13% | 0.155% | -51.13% | 1049 |
| 80 | $5,274.50 | 27.099 | 79.049 | 0.336% | -50.94% | 0.132% | -58.49% | 1050 |
| 100 | $5,247.92 | 27.100 | 79.052 | 0.292% | -57.36% | 0.113% | -64.57% | 1051 |

Abbreviations: LB-B (liquid biopsy with high sensitivity for local cancers and sensitivity for high-grade dysplasia); GC (gastric cancer); HGD (high-grade dysplasia); QALY (quality-adjusted life-years); EGD (esophagogastroduodenoscopy).

^a^Percent averted is calculated relative to the base-case natural history scenario.

**Table S5. Two-way sensitivity analysis varying liquid biopsy (LB-B) specificity and HGD detection.**

| **Specificity Level %** | **Sensitivity for HGD %** | **Total Cost** | **QALYs** | **Life Expectancy** | **GC Incidence** | **% averted^a^** | **GC Mortality** | **% averted^a^** | **Lifetime EGDs per 1000** |
| --- | --- | --- | --- | --- | --- | --- | --- | --- | --- |
| 0 | 0 | $8,740.17 | 27.096 | 79.045 | 0.379% | -44.63% | 0.161% | -49.48% | 9453 |
| 0 | 0.2 | $8,703.71 | 27.097 | 79.048 | 0.316% | -53.74% | 0.133% | -58.09% | 9457 |
| 0 | 0.4 | $8,675.37 | 27.098 | 79.051 | 0.269% | -60.63% | 0.112% | -64.70% | 9460 |
| 0 | 0.6 | $8,652.79 | 27.098 | 79.053 | 0.233% | -65.98% | 0.096% | -69.91% | 9462 |
| 0 | 0.8 | $8,634.42 | 27.099 | 79.055 | 0.203% | -70.26% | 0.082% | -74.12% | 9464 |
| 0 | 1 | $8,619.20 | 27.099 | 79.056 | 0.179% | -73.75% | 0.071% | -77.58% | 9466 |
| 0.2 | 0 | $7,996.41 | 27.096 | 79.043 | 0.424% | -38.00% | 0.179% | -43.80% | 7562 |
| 0.2 | 0.2 | $7,955.58 | 27.097 | 79.046 | 0.354% | -48.30% | 0.148% | -53.45% | 7566 |
| 0.2 | 0.4 | $7,923.75 | 27.098 | 79.049 | 0.300% | -56.11% | 0.124% | -60.88% | 7568 |
| 0.2 | 0.6 | $7,898.34 | 27.099 | 79.052 | 0.258% | -62.21% | 0.106% | -66.75% | 7571 |
| 0.2 | 0.8 | $7,877.64 | 27.099 | 79.054 | 0.225% | -67.09% | 0.091% | -71.50% | 7572 |
| 0.2 | 1 | $7,860.47 | 27.100 | 79.055 | 0.198% | -71.07% | 0.078% | -75.40% | 7574 |
| 0.4 | 0 | $7,255.36 | 27.095 | 79.040 | 0.477% | -30.30% | 0.199% | -37.25% | 5672 |
| 0.4 | 0.2 | $7,209.54 | 27.097 | 79.045 | 0.397% | -41.97% | 0.165% | -48.09% | 5675 |
| 0.4 | 0.4 | $7,173.72 | 27.098 | 79.048 | 0.336% | -50.85% | 0.138% | -56.46% | 5677 |
| 0.4 | 0.6 | $7,145.08 | 27.099 | 79.051 | 0.289% | -57.80% | 0.117% | -63.09% | 5679 |
| 0.4 | 0.8 | $7,121.70 | 27.099 | 79.053 | 0.251% | -63.37% | 0.100% | -68.46% | 5681 |
| 0.4 | 1 | $7,102.29 | 27.100 | 79.055 | 0.219% | -67.92% | 0.086% | -72.88% | 5682 |
| 0.6 | 0 | $6,517.62 | 27.095 | 79.038 | 0.538% | -21.27% | 0.224% | -29.63% | 3782 |
| 0.6 | 0.2 | $6,466.05 | 27.096 | 79.042 | 0.448% | -34.53% | 0.185% | -41.84% | 3785 |
| 0.6 | 0.4 | $6,425.65 | 27.098 | 79.046 | 0.379% | -44.65% | 0.155% | -51.30% | 3787 |
| 0.6 | 0.6 | $6,393.26 | 27.099 | 79.049 | 0.324% | -52.59% | 0.131% | -58.80% | 3789 |
| 0.6 | 0.8 | $6,366.79 | 27.099 | 79.052 | 0.281% | -58.96% | 0.112% | -64.89% | 3790 |
| 0.6 | 1 | $6,344.78 | 27.100 | 79.054 | 0.245% | -64.19% | 0.096% | -69.91% | 3791 |
| 0.8 | 0 | $5,783.91 | 27.095 | 79.035 | 0.612% | -10.54% | 0.252% | -20.68% | 1894 |
| 0.8 | 0.2 | $5,725.70 | 27.096 | 79.040 | 0.508% | -25.66% | 0.208% | -34.48% | 1896 |
| 0.8 | 0.4 | $5,679.97 | 27.098 | 79.044 | 0.429% | -37.24% | 0.174% | -45.20% | 1898 |
| 0.8 | 0.6 | $5,643.23 | 27.099 | 79.047 | 0.367% | -46.35% | 0.147% | -53.73% | 1899 |
| 0.8 | 0.8 | $5,613.14 | 27.099 | 79.050 | 0.317% | -53.68% | 0.125% | -60.66% | 1900 |
| 0.8 | 1 | $5,588.10 | 27.100 | 79.052 | 0.276% | -59.69% | 0.107% | -66.39% | 1901 |
| 1 | 0 | $5,055.18 | 27.094 | 79.032 | 0.700% | 2.34% | 0.286% | -10.06% | 8 |
| 1 | 0.2 | $4,989.24 | 27.096 | 79.037 | 0.582% | -14.97% | 0.236% | -25.71% | 9 |
| 1 | 0.4 | $4,937.26 | 27.097 | 79.042 | 0.491% | -28.28% | 0.197% | -37.91% | 10 |
| 1 | 0.6 | $4,895.42 | 27.098 | 79.045 | 0.419% | -38.77% | 0.166% | -47.65% | 10 |

**Table S5. Two-way sensitivity analysis varying liquid biopsy (LB-B) specificity and HGD detection. (continued)**

| **Specificity Level %** | **Sensitivity for HGD %** | **Total Cost** | **QALYs** | **Life Expectancy** | **GC Incidence** | **% averted^a^** | **GC Mortality** | **% averted^a^** | **Lifetime EGDs per 1000** |
| --- | --- | --- | --- | --- | --- | --- | --- | --- | --- |
| 1 | 0.8 | $4,861.09 | 27.099 | 79.049 | 0.361% | -47.24% | 0.141% | -55.57% | 11 |
| 1 | 1 | $4,832.46 | 27.100 | 79.051 | 0.313% | -54.20% | 0.120% | -62.13% | 11 |

Abbreviations: LB-B (liquid biopsy with high sensitivity for local cancers and sensitivity for high-grade dysplasia); GC (gastric cancer); HGD (high-grade dysplasia); QALY (quality-adjusted life-years; EGD (esophagogastroduodenoscopy).

^a^Percent averted is calculated relative to the base-case natural history scenario.
